# Identification of residues for chaperone-like activity of OppA protein in *Yersinia pseudotuberculosis*

**DOI:** 10.1186/s13568-020-01090-8

**Published:** 2020-08-21

**Authors:** Elena Escobar Garduño, Thomas Scior, Lucia Soto Urzúa, Luis Javier Martínez Morales

**Affiliations:** 1 Centro de Investigaciones en Ciencias Microbiológicas, Instituto de Ciencias, IC-11 CU San Manuel, Benemérita Universidad Autónoma de Puebla, Puebla, México; 2 Facultad de Ciencias Quimicas, Benemérita Universidad Autónoma de Puebla, C.U. Av. Sn. Claudio y 24 sur Col. Sn. Manuel, C.P.72570 Puebla, Pue México

**Keywords:** α-glucosidase, Lactate dehydrogenase, OppA, Site-directed mutagenesis, Analogy modeling, Chaperone-like activity

## Abstract

Periplasmic oligopeptide binding protein (OppA) is part of a multimeric cytoplasmic membrane protein complex, whose function is known as peptide transporters found in Gram-negative bacteria. A chaperone-like activity has been found for the OppA from *Yersinia pseudotuberculosis*, using biochemical experiments. Through computational analysis, we selected two amino acid residues (R41 and D42) that probably are involved in the chaperone-like activity. Our results to corroborate how OppA assists refolding and renaturation of lactate dehydrogenase and alpha-glucosidase denatured enzymes.

## Introduction

The pentameric OppA/B/C/D/F is part of a huge protein ATP-binding cassette (ABC) system of related transporters to take up peptides into the cells. The complex embraces at the extracellular side our target protein OppA, which itself is associated with two pore-forming transmembrane proteins (OppB and OppC) as well as two nucleotide-binding proteins (OppD and OppF) which function as ATPases (Monnet 2003). OppA binds to an incoming oligopeptide and delivers it to the import complex in the inner membrane, which requires ATP hydrolysis to transport the oligopeptide into the cell. The Opp multi-subunit system is involved in nutrient uptake, signaling processes (including regulation of gene expression), development competence, sporulation, DNA transfer by conjugation, virulence development, as well as chemotaxis (Garmory and Titball 2004). Precisely, the OppA protein possess versatility in substrate recognition, reflecting the cavernous architecture at the binding site (Tanabe et al. 2007). This broad specificity suggests that the Opp system might be exploited for the uptake of novel antimicrobials. The OppA protein is also immunogenic, it has been proven that the *Yersinia pestis* OppA protein provides protection against experimental plague in mouse models (Tanabe et al. 2006).

Upon genetical manipulations Lennon and coworkers found the periplasm enriched with our target OppA among others like DppA, Ivy, OsmY or HdeB. So, the researchers could test the chaperone-like activity in vitro with positive results for OppA and closely related DppA (Lennon et al. 2015).

The *Escherichia coli* oligopeptide binding protein OppA binds to peptides between two and five amino acids, and has preference for basic peptides composed by lysine residues (Klepsch et al. 2011). The crystal structure of the *Yersinia pestis* OppA protein (Tanabe et al. 2007) (PDB code: 2Z23), shows that electrostatic interactions are pivotal for the association between certain **p**eriplasmic **b**inding **p**roteins (PBPs) and their specific transmembrane domain (TMDs). For OppA, and possibly other PBPs, it is likely that atomic interactions involving *Van der Waals* forces and hydrogen bonding constitute essential features for substrate recognition (Tanabe et al. 2007).

**Fig. 1:**
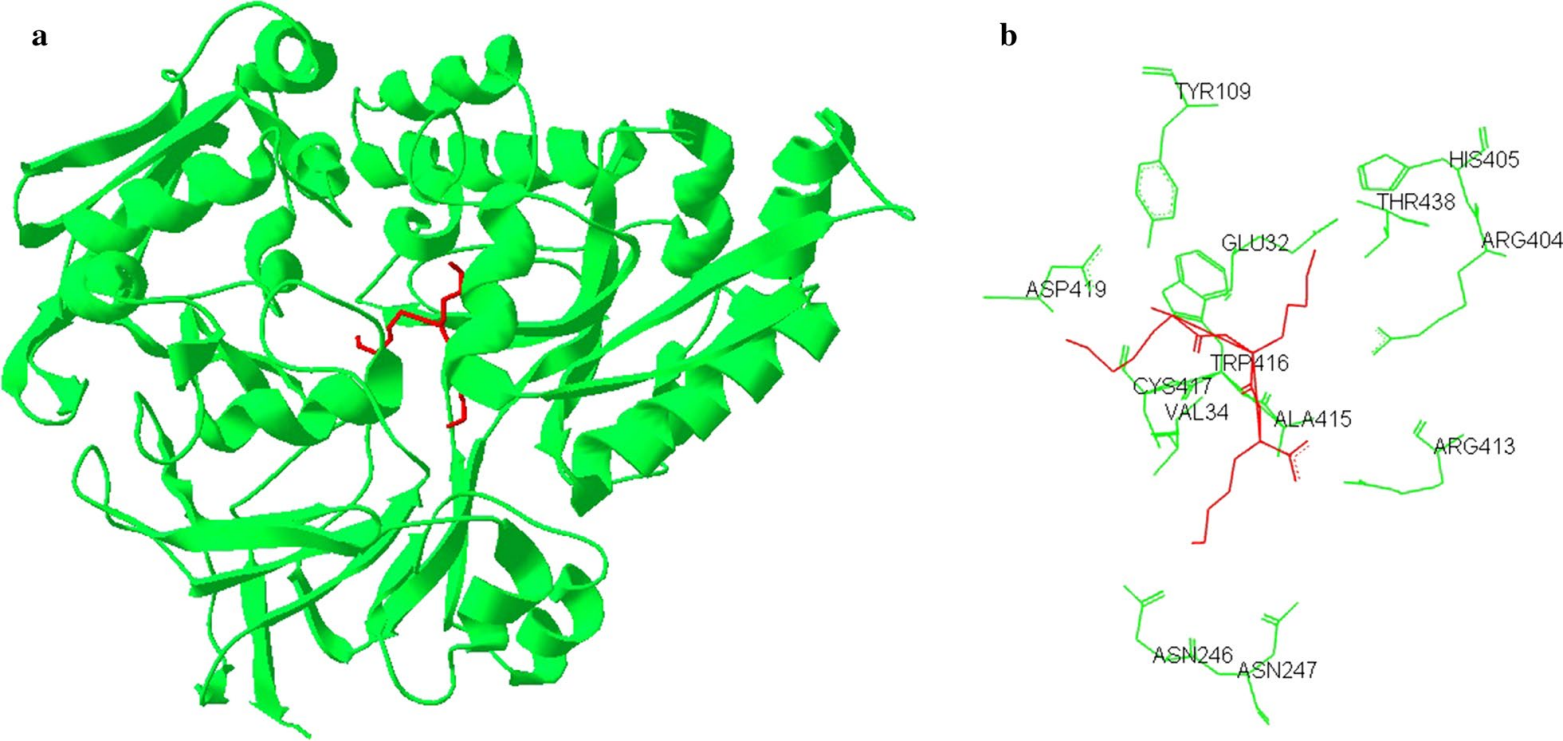
Crystal structure of *Y. pestis* OppA protein. The OppA protein (green) is bound to tri-lysine peptide (red). PDB code: 2Z23. **a** The OppA protein is formed by two lobes with secondary structural βαβ units and a flexible hinge between the two lobes where the substrate-binding cleft is located. The ligand is completely enclosed in the hydrophobic cleft. **b** Key binding-site residues are labeled (residues Glu32, Val34, Tyr109, Asn246, Asn247, Arg404, His405, Arg413, Ala415, Trp416, Cys417, Asp419, and Thr438). Modified from (Tanabe et al. 2007)

The liganded crystal structure of *Y. pestis* OppA has been elucidated in the closed state, i.e. in complex with a short tripeptide (Tanabe et al. 2007) (Fig. 1). This Lys-Lys-Lys ligand has become an experimental test substance to probe conformational preferences, a pre-requisite for the study of unfolded and refolded states which were found to be nonrandom, i.e. no random coil state is acquired with conformations found in all space-accessible parts of the torsional phi-psi plots (Verbaro et al. 2012).

Other liganded crystal complexes show the same closed state as *Y. pestis* where the two flanking domains (bilobate) linked by a hinge region bury the ligand in a deep cleft, e.g. *Salmonella typhimurium* OppA and *Escherichia coli* OppA (Klepsch et al. 2011). In order to study the exceptionally wide range of ligand structures recognized by OppA (Tame et al. 1995; Berntsson et al. 2009), crystal complexes were computationally simulated to analyze nonbonded interactions of main and side chains and water dissolvation effects with twenty eight tripeptides with a Lys-Any-Lys pattern (Tian et al. 2011).

The domain rotations allow flexible conformational rearrangements that reflect the discussed sequence-independent substrate recognition engaging a so-called hinge-region between flanking domains.

Other important periplasmic proteins are periplasmic chaperones. Some of these proteins have dual activity, such as SurA (Soltes et al. 2016), PpiD (Matern et al. 2010), and FkpA (Arié et al. 2001), which also act as peptidyl-prolyl *cis*-*trans* isomerases and DegP (HtrA) (Krojer et al. 2002) that has protease activity. In addition to the periplasmic chaperones mentioned above, a seminal work in 1997 reported on chaperone-like activities for *E. coli* in the case of OppA, as well as MalE (maltose-binding protein E) (Richarme and Caldas 1997). Matsuzaki et al. (1998) detected a protein with chaperone-like activity in the periplasm. This periplasmic protein constitutes the dipeptide-binding protein (DppA) because it imports dipeptides from the outside. Of note, DppA is structurally and functionally related to target OppA. Intriguingly, chaperone-like activities were reported for DppA, but not for OppA, which also hint at the function of chaperonin GroEL from *E. coli* (Matsuzaki et al. 1998, 2000). It was shown that aggregation of (unfolded substrate) protein dimethyl sulfoxide reductase (DMSOR) meant that no folding took place. Inversely, renaturation of unfolded DMSOR would prevent aggregation. Disrupting the gene of *dpp*A did not alter active DMSOR formation in *Rhodobacter sphaeroides dpp*A^*−*^ bacteria due to the presence of DctP and BztA which acted as periplasmic chaperone-like proteins (Matsuzaki et al. 2000). Hence, this research group concluded that several proteins were necessary for DMSOR refolding in the periplasm (Matsuzaki et al. 2000).

In *Yersinia pseudotuberculosis*, the OppA protein is localized in the periplasm and it has been determined by renaturation assays of the heat-denatured α-glucosidase enzyme that OppA has chaperone-like activity (data obtained from our laboratory). The relationship between the proposed dual activity of the *Y. pseudotuberculosis* OppA protein is not clearly analyzed, so in this study an amino acid substitution was performed by site-directed mutagenesis to determine how these changes affect chaperone-like activity in OppA.

Our experiments aimed at elucidating a hitherto poorly understood role of OppA from *Y. pseudotuberculosis* in the refolding process of cell proteins, in contrast to its well-known role as an ABC transporter component. To this end, we chose two unrelated target proteins for our in vitro refolding tests in the presence of OppA after chemical denatutation. The target proteins were α-glucosidase; α-GLD and lactate dehydrogenase; LDH, which do not share any structural or functional features. To identify those OppA residues which assist its role in refolding, we carried out mutational changes of the primary sequence of OppA.

## Materials and methods

### Construction of pEXP-htrA plasmid

The *htr*A (> CP009792.1:4,438,735–4,440,180) gene was obtained by PCR amplification using the pCHTRA7 plasmid as a template (unpublished results). This plasmid is derived from pCR2.1TOPO (Invitrogen, USA) which have cloned the *htr*A gene, 457 base pairs (bp) upstream and 412 bp downstream of the *Y. pseudotuberculosis* YPIII *htr*A gene. The primers used for the amplification were: htrATG_mut_del 5′ATG AAA AAA ACC ACG TTAG and htrA_CTG_rev 5′CTG CAT CAA TAA ATA GAG TG 3′, using Taq Platinum from Invitrogen®. The 1 444 pb PCR product was cloned into the pEXP5-CT/TOPO (Invitrogen, USA) vector. Random selected clones were digested with *Bgl*II and *Eco*RI, and clones with the restriction pattern expected were sequenced.

### Site-directed mutagenesis

The *opp*A gene (> CP009792.1:2,946,314–2,947,951) is cloned into the pEXP5-CT expression vector, pEXP-oppA (unpublished results), the construction was used as a DNA template for inverse PCR. In order to obtain mutant R41A:D42A, two mutagenic primers were designed following the instructions from the QuickChange II (Agilent, USA) method. Primers BsgI_mut02_del 5′CCTGAATCCAATATTTCA**CG**TC**CA**CTACTTGAAGGGCTGGTG and BsgI_mut02_rev 5′CACCAGCCCTTCAAGTAG**TG**CA**GC**TGAAATATTGGATTCAGG (in bold, the nucleotides changed to generate the amino acid substitution), two recognition sites for restriction enzymes *Bsg*I and *Pvu*II (underlined) were also included. *Pvu*II was used to select the clones that had the desired mutation.

To obtain the OppAD419G:Y420G and OppAR41A:D42A/D419G:Y420G mutants, an inverse PCR was performed using the plasmid pEXP-OppA as a template for mutant OppAD419G:Y420G and plasmid pEXP-OppAR41A:D42A for mutant OppAR41A:D42A:D419G:Y420G. For this inverse PCR we used two primers designed back to back and phosphorylated at the 5′end, in this system only one primer is mutagenic. The primers used were: 01__del / 5Phos / GC TTG GTG TGC **A**G**G**T**GG** CAA TGA GCC ATC CTC CTT CCT AAA TAT G (nucleotides changed to replace amino acids D419G / Y420G are in bold) and 01_rev / 5Phos / GG CCC GGG CAA CAT CAT AAG TCC CCT GGT GAC GGG TAT CC (the cut site for the *Sma*I enzyme is underlined). The PCR product was digested by the enzyme *Dpn*I, after which it was self-circularized by ligation reaction using the enzyme T4 DNA Ligase Thermo Scientific® (Thermo Fisher Scientific, USA). It was subsequently transformed into chemically competent TOP10 cells and plated on LB agar supplemented with Amp 100 µg/mL, the selection was the same as that used for the OppAR41A:D42A mutant. Random clones were selected and its plasmids were digested with *Sma*I, the clones with the expected restriction pattern were selected for sequencing.

### Purification of proteins

Plasmids pEXP-OppA, pEXP-OppAR41A:D42A, pEXP-OppAD419G:Y420G, pEXP-OppAR41A:D42A/D419G:Y420G, and pEXP-htrA were transformed into *E. coli* BL21 (pLys). Cells harboring these plasmids were grown in LB medium supplemented with Amp 100 µg/mL and incubated at 37 °C with constant stirring. At an O.D._600_ of 0.5, IPTG (Invitrogen, USA) was added to the cultures at a final concentration of 0.15 mM. After three hours of induction, cells were harvested, washed with PBS buffer, and frozen at − 70 °C overnight. The pellet was resuspended in lysis buffer (20 mM KH_2_PO_4_, 250 mM KCl, 20 mM imidazole, 1 mg/mL of lysozyme pH 7.0) and incubated on ice for 30 min. Then, *N*-lauroyl-sarcosine was added to a final concentration of 0.1% and the mix was incubated on ice for 30 min more. The suspension was then sonicated and centrifuged at 5000 rpm for 15 min, the supernatant was dialyzed in 20 mM KH_2_PO_4_, 250 mM KCl, pH 7 buffer (buffer A). After that, the bacterial extracts were loaded onto a Sephadex G-50 column equilibrated in 20 mM KH_2_PO_4_, 250 mM KCl pH 7 buffer, and eluted in the same buffer. The fractions were analyzed by 10% SDS-polyacrylamide gel to determine which fractions contain proteins, these fractions were allowed to interact with Ni-NTA resin for 2 h at 4 °C with constant stirring. After that, protein purification was performed following the manufacturer’s protocol for protein purification in native conditions. In order to eliminate imidazole, proteins were equilibrated in 20 mM KH_2_PO_4_, 250 mM KCl buffer pH 7 by gel filtration chromatography on a Sephadex G-50 column. Fractions were quantified by Bradford assay and immediately used for the chaperone activity assays.

### Chaperone-like activity assays

#### α-glucosidase renaturation assay

Urea in a concentration of 4 m at 20 °C was the means of denaturing 8.4 µg of α-glucosidase (α-Glucosidase from *Saccharomyces cerevisiae*, Sigma-Aldrich, USA), during 20 min, at 25 °C. After that, 100 µl of 50 mM KH_2_PO_4_, 200 mM KCl, pH 7 buffer was added, then 39 µg of bovine serum albumin (BSA), 26.7 µg of HtrA, 34 µg of OppA proteins were added in each treatment, finally were added 50 µl of protease inhibitor 10X and 50 mM KH_2_PO_4_, 200 mM KCl, pH 7 untill 500 µl of final volume mix. The treatments were: (i) the negative control was BSA in a 1150 nM concentration, (ii) target OppA was prepared in a concentration of 1150 nM, and (iii) the positive control HtrA was prepared in a 1000 nM concentration (1 µM). The SIGMAFAST™ (Sigma, USA. Cat. S8830) Protease Inhibitor reactant was added according to the protocol of the commercial kit.

The molar α-glucosidase: OppA molar ratio was 1:4, while the urea concentration during the refolding process was held at 80 mM, because at this concentration the activity of the enzyme α-glucosidase is not affected which was empirically determined.

In the following step, all the treatments were incubated at room temperature during one and a half hours and subsequently 2 µg maltose sugar was added and the mixture was stirred to obtain a solution. Finally, after another hour and a half the glucose concentration was quantified by standard glucose oxidase/peroxidase protocol from BioSystems® (Ref. 11,504). The tests were performed in triplicate for statistical proof.

#### Lactate dehydrogenase renaturation assay

1.25 µg of lactate dehydrogenase from rabbit muscle (Sigma-Aldrich®, Sigma, USA) was denatured with 1 molar guanidine hydrochloride (GdnHCl) during 10 min at 25 °C. After that, 100 mM of NaH_2_PO_4_ pH 7 buffer was added, the final volume mix was 500 µl. The mix was incubated at 25 °C for 90 min with 287 nM chaperones: BSA (negative control), OppAWT, OppA mutants and HtrA (as positive control). Again, The SIGMAFAST™ (Sigma, USA) Protease Inhibitor was added according to the protocol of the commercial kit. Lactate dehydrogenase (71.43 nM) activity assays were performed at 25 °C according to Sigma-Aldrich® (Sigma, USA) protocol. β-NADH was measured by spectrophotometry at 340 nm every 10 s for 5 min. LDH units in each assay were calculated according to LDH activity assay protocol from Sigma-Aldrich® (Sigma, USA).

The molar ratio beween LDH and chaperone-like proteins (OppA, BSA and HtrA) was set to the same proportion as before, 1 to 4, while GdnHCl was applied at a 20 mM concentration because at this concentration level the LDH activity remains unaltered, all of which had been empirically determined prior to our bioassays.

### Statistical analysis

All the measurements were carried out in triplicate and results were expressed in terms of mean values ± standard deviation whereas the corresponding percentage of enzymatic activity values were calculated using statistical software. (Sigma Plot for Windows versión 10.0. Systat software, Inc., Germany). All tests were performed in triplicate for statistical proof applying One way ANOVA.

## Results

### Residue selection for mutational studies

In order to determine the amino acids to be substituted, an in silico comparison of the crystallographic coordinates for three *E. coli* proteins was carried out: (i) OppA in an open conformation without ligand (PDB code: 3TCH (Klepsch et al. 2011), (ii) in a closed conformation with bound Arg-Gly-Glu tripeptide (PDB code: 3TCG (Klepsch et al. 2011), and (iii) with periplasmic chaperone HdeA (PDB code: 1DJ8 (Gajiwala and Burley 2000). Molecular modeling was performed under Swiss-Pdb-Viewer (Guex and Peitsch 1997) and Discovery Studio free visualizer (Dassault Systemes B. [Internet]. 2016). The latter was selected because it is a known 3D structure with experimentally demonstrated chaperone-like activity. It is also a small single-chained protein with alpha helical stability like OppA. Since its activity was observed without doubts it is a reliable source of atomistic insight to substrate recognition upon folding reported in the original work (Gajiwala and Burley 2000).

**Fig. 2:**
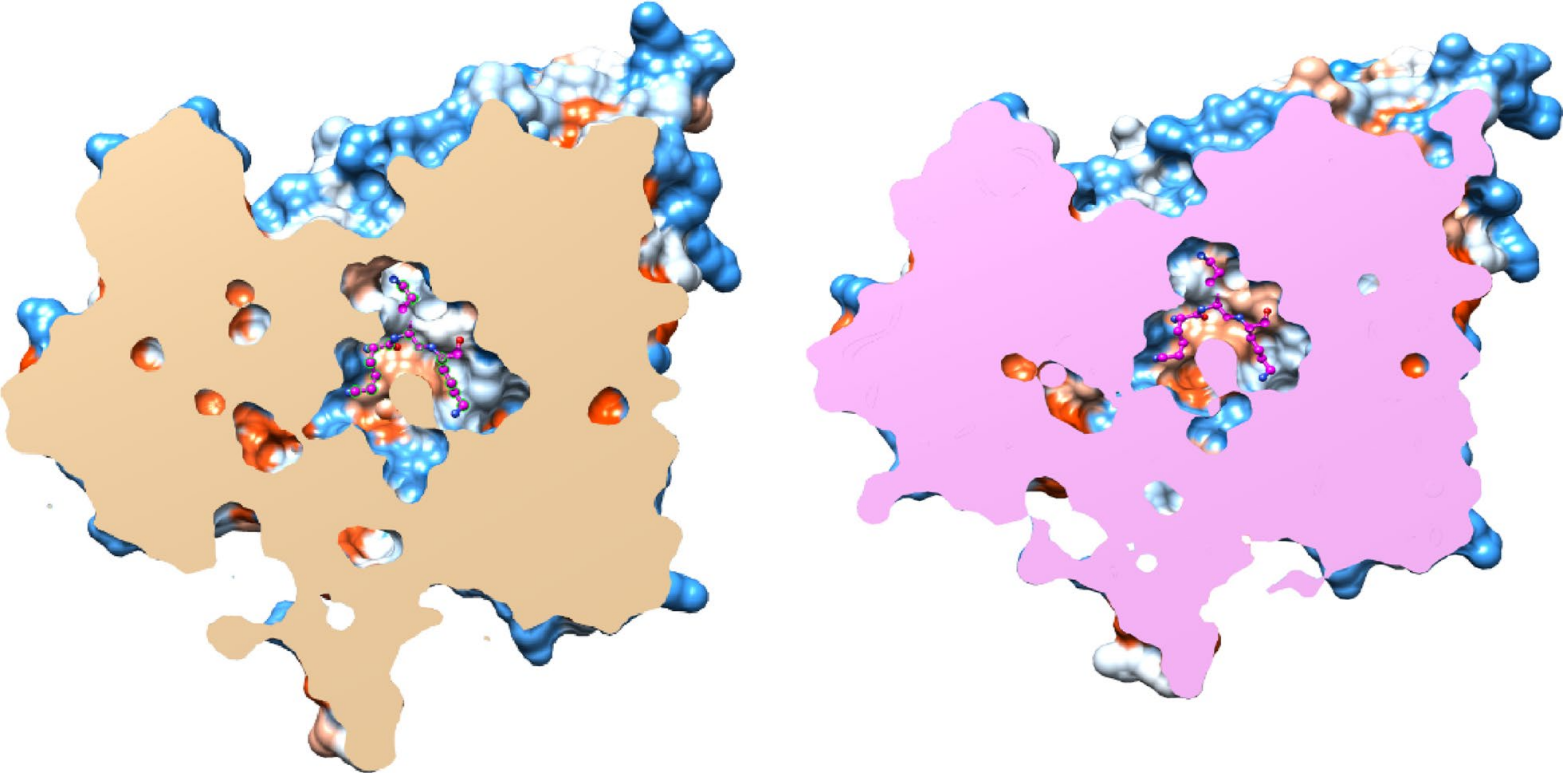
Insight view into the protein cavities of OppA in open and closed states (left/right PDB codes: 3TCG/2Z23 from *E. coli*/*Y. pestis*) to distinguish the conformational rearrangements. The tri-lysine ligand (from PDB code: 2Z23) was also merged into the other binding site (of PDB code: 3TCG, central hole). Color code for protein surface: bluish/white7redish for positive/neutral/negative partial atom charges, respectively

Prior to selecting the residues to be replaced, a possible mechanistic role for refolding mechanism was discussed for heat shock proteins (HSP) in general, and especially the case of small HSPs (sHSP) (Skorko-Glonek et al. 2007; Matsuzaki et al. 2000), PDB code: 5D4W (Heuck et al. 2016) and PDB code: 3AAC (Takeda et al. 2011). To this regard, the open and closed conformational states were also inspected for the crystal structure of *E. coli* OppA complexed with the Arg-Gly-Glu tripeptide (Fig. 2) (PDB codes: 3TCH and 3TCG (Klepsch et al. 2011).

**Fig. 3:**
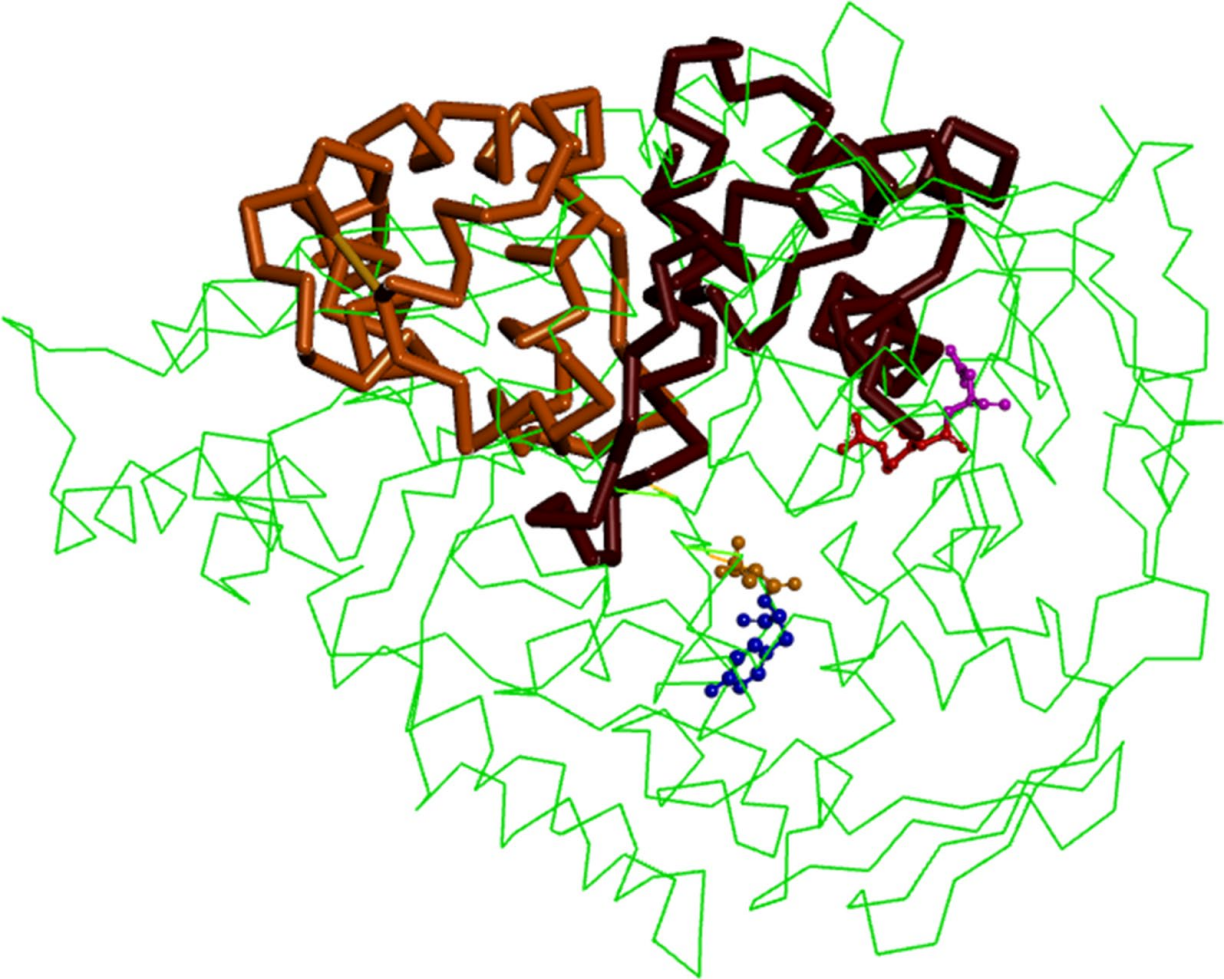
Overlay of *Yersinia pestis* OppA PDB:2Z23 (green color) and HdeA PDB:1DJ8 (in light brown color the monomer **a**, in dark brown color the monomer **b**). The amino acids arginine 41 (red) and aspartate 42 (pink) affect the protein surface near to the hydrophobic cleft; amino acids aspartate 419 (orange) and tyrosine 420 (blue) are in the oligopeptide binding cleft

**Fig. 4:**
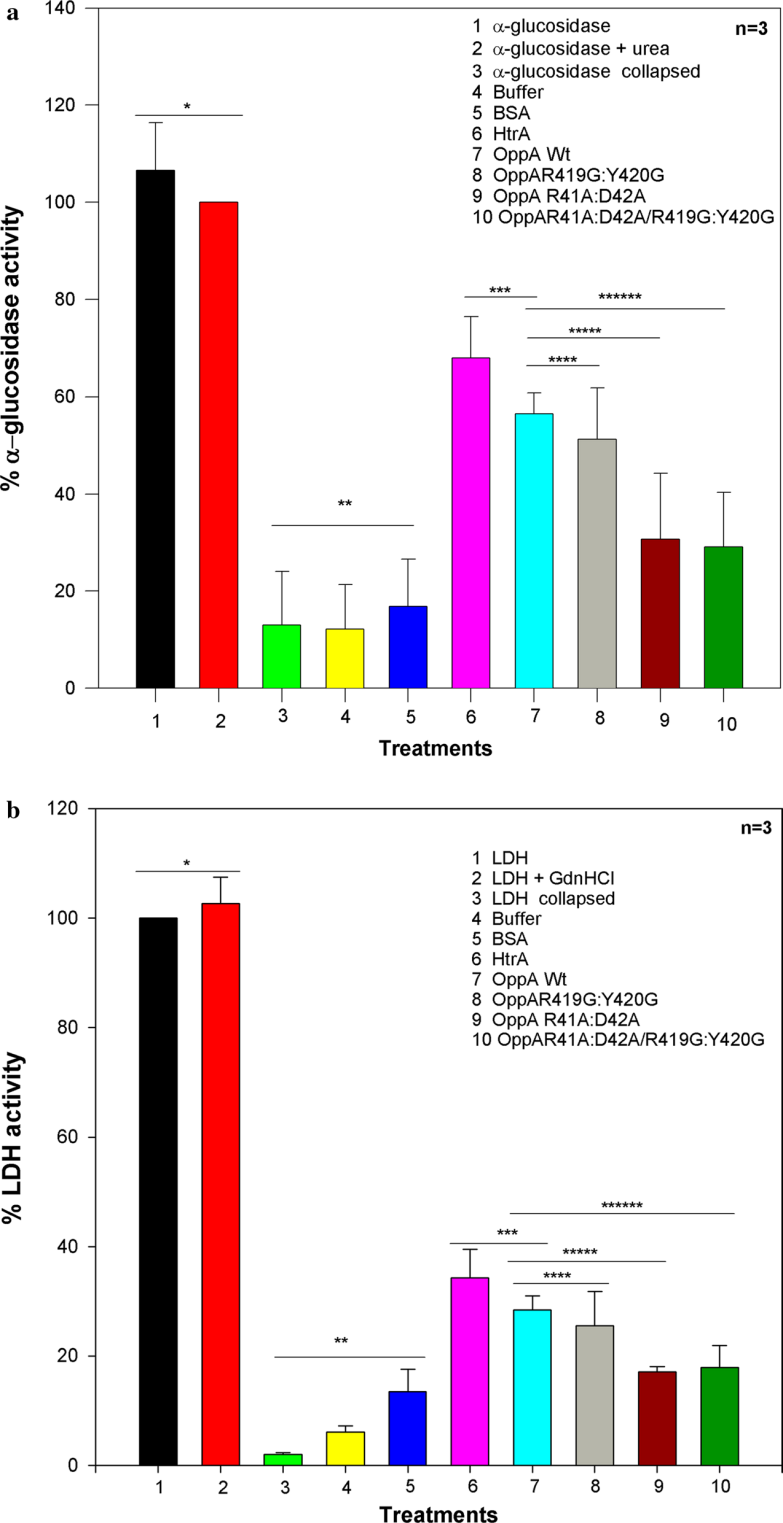
Enzymatic activities after denaturation with chaotropic agents and renaturation with different proteins. The collapsed enzyme was denatured with the chaotropic agents and was not subjected to renaturation process and serves as negative control. **a** Percent of α-glucosidase activity measured after denaturation and renaturation assays. α-glucosidase enzyme was denatured with urea (80 mM) and renatured under different treatments, 1 for α-glucosidase; 2 for α-glucosidase + urea; 3 for collapsed α-glucosidase; 4 for Buffer; 5 for BSA; 6 for HtrA; 7 for wild type OppA; 8 for mutant type OppA with double mutation R419G & Y420G; 9 for mutant type OppA with double mutation R41A & D42A; and finally, 10 for mutant type OppA with both double mutations R41A & D42A and R419G & Y420G. The more significant data are shown, HtrA vs. OppAWt with *p* = 0.104, OppAWt vs. OppA R419G:Y420G with *p* = 0.472, OppAWt vs. OppA R41A:D42A with *p* = 0.035, OppAWt vs. OppA R41A:D42A / R419G:Y420 with *p* = 0.017. **b** Percentage of LDH activity measured after denaturation and renaturation assays. Lactate-dehydrogenase enzyme was denatured by 1.25 molar guanidine hydrocloride and renatured under different conditions, 1 for LDH; 2 for LDH + GdnHCl; 3 for collapsed LDH; 4 for Buffer; 5 for BSA; 6 for HtrA; 7 for wild type OppA; 8 for mutant type OppA with double mutation R419G & Y420G; 9 for mutant type OppA with double mutation R41A & D42A; and finally, 10 for mutant type OppA with both double mutations R41A & D42A and R419G & Y420G. The more significant data are documented, HtrA vs. OppAWt with *p* = 0.148, OppAWt vs. OppA R419G:Y420G with *p* = 0.505, OppAWt vs. OppA R41A:D42A with *p* = 0.002, OppAWt vs. OppA R41A:D42A / R419G:Y420 with *p* = 0.0193. The data were analyzed statistically by One-Way ANOVA using Sigma Plot®. The values correspond to the results of three independent repetition assays. Of note, for a detailed interpretation of the result see the main text above

The periplasmic chaperone HdeA (Fig. 3) was used as a reference protein to pinpoint the selected amino acids for mutational studies. HdeA is a homodimeric protein in its inactive state and monomeric in its active conformation. HdeA was superimposed on the open and closed conformation structures of OppA from *E. coli* and *Y. pestis*, and we selected the amino acids Arg 41 and Asp 42 to replace them by alanine. The amino acids Asp 419 and Tyr 420 were mutated to D419G and Y420G (Fig. 4).

### Chaperone-like activity

#### α.glucosidase renaturation assay

The α-glucosidase enzyme was denatured and renatured (as described in methods) using a buffer, BSA, HtrA, OppA WT, and OppA mutant proteins. The rate of activity recovery in renaturation buffer 50 mM KH_2_PO_4_ and 200 mM KCl was 13.6%, with BSA 25.5%, with HtrA 68%, with OppA WT 56.4%, with OppAD419G:Y420G 51.2%, with OppA R41A:D42A 30.7%, and with OppAR41A:D42A:D419G:Y420G 29.1% compared to non-denatured α-glucosidase in buffer A with 0.08 m urea (Fig. 4a). These data suggest that the OppA protein has chaperone-like activity on α-glucosidase under the tested conditions. To draw final conclusions the results had to undergo a systematic interpretation. To this end, we designed a total of ten assay constellations varying the composition of each test. Precisely, all experimental data were statistically analyzed and plotted in a histogram for direct comparison (Fig. 4). All data were subjected to statistical significance tests by Fisher’s p-values and listed (Table 1). As a direct result of the comparison between wild type 7 versus mutant types 8, 9 or 10 it became evident that the latter two mutations of OppA (9, 10) significantly affect chaperone activity. This finding is also reflected in the histograms (Fig. 4).

**Table 1:** P-values

Treatments	p-values (α-glucosidase)	p-values (LDH)
1, 2	0.700	0.700
3, 4, 5	0.837	0.004
(1, 2) vs. (3, 4, 5)	0.001	0.011
3, 4, 5	0.837	0.004
6, 7, 8, 9, 10	0.003	0.002
(3, 4, 5) vs. (6, 7, 8, 9, 10)	0.001	0.001
6,7	0.104	0.148
7, 8	0.472	0.505
7, 9	0.035	0.002
7, 10	0.017	0.019
5, 6	0.002	0.005
5, 7	0.003	0.006

Table 1 P-values: Listing of statistical data for α-glucosidase and LDH essays in 2nd and 3rd columns, respectively. The first column lists the sample groups or treatments The ten treatments were labelled 1 through 10 with 1 for α-glucosidase; 2 for α-glucosidase + urea; 3 for collapsed α-glucosidase; 4 for Buffer; 5 for BSA; 6 for HtrA; 7 for wild type OppA; 8 for mutant type OppA with double mutation R419G & Y420G; 9 for mutant type OppA with double mutation R41A & D42A; and finally, 10 for mutant type OppA with both double mutations R41A & D42A and R419G & Y420G. “1” to “10”. Statistically significant differences are achieved between the samples with p-values much smaller than 0.05 (Fisher’s p-values).

Precisely, this statistically significant difference in the activities between undenatured and denatured (i.e. without refolding process) enzymes is a key finding here. The comparison between treatments 5 and 6 unveil the statistically significant difference with p-value of 0.002, all of which clearly hints at HtrA’s refolding activity. The treatments 3, 4 and 5 also differ significantly from 6, 7, 8, 9 and 10 with p = 0.003. This comparison demonstrated that the proposed proteins had effectively worked as refoldases. In addition, it is safe to utter that treatment 7 is significantly different from treatments 9 (p < 0.035) or 10 (p < 0.017) because both are lying under the significance test threshold of 0.05. Both results show that the corresponding mutations of OppA actually do affect the chaperone activity under scrutiny. Doubts remain, however, for the interpretation of treatments 7 and 8 (p < 0.472). Even without having successfully passed the test for statistical significance, the ultimate decision to get knowledge about the biochemical significance is the experiment and hence it cannot be ruled out that treatments 7 with 8 affect the chaperone activity in the same way as both statistically well-behaving pairs of 7 with 9 and 7 with 10 do. Since the measured activity values depend on the fact whether or not a refolding process of enzymes took place and to what degree the protein could refold. An incomplete refolding process can reflect a poor or even missing statistical significance of activity difference. That said, it seems not far-fetched to assume that a partial—and not a complete refolding—is exactly what happened to the treatments 7 with 8 here.

#### LDH renaturation assay

The rate of LDH activity recovery in buffer B was 6.04%, with BSA 13.4%, with HtrA 34.3%, with OppA WT 28.4%, with OppAD419G:Y420G 25.52%, with OppAR41A:D42A, 17%, and with OppAR41A:D42A:D419G:Y420G 17.8% compared to non-denatured LDH in buffer B with 0.025 m Gdn-HCl (Fig. 4b). Details about the compared treatments and the statistical results are displayed in panel B of Fig. 4. The same numbering scheme was applied in Panel B as in Panel A of Fig. 4 to ease the direct comparison of all ten treatments. The following outlines the key aspects for the LDH assays. Upon comparison of treatments 1 and 2 against 3, 4 and 5 a significant difference with p-value of 0.011 was achieved. Hence, it can be concluded that the statistically significant test was passed successfully. The difference in enzymatic activities hints at the undenatured and denatured states of proteins, i.e. the measured activity values depend on the fact whether or not a refolding process of enzymes took place. The comparison between treatments 5 and 6 showed the statistically significant difference with p = 0.006 demonstrating HtrA’s successful refolding process. The difference between treatments 3, 4 and 5 against 6, 7, 8, 9 and 10 were significant without doubts (p = 0.002). The result demonstrated that the proteins under study had effectively worked as refoldases. In addition, treatment 7 can be compared with treatment 8 (p < 0.505), or with treatment 9 (p < 0.002), and also with treatment 10 (p < 0.019) to show that certain mutations of OppA actually do affect the chaperone activity under scrutiny. Again, as with the α-glucosidase assays, the relative significance test failure of treatment 7 and 8 (threshold 0.05 < p < 0.505) indicates that refolding took not place in a perfect way to reconstitute plain enzymatic activity of the target.

## Discussion

We tested the ability of the OppA protein to refold proteins denatured with chaotropic agents such as guanidine-hydrochloride and urea. Our data suggest that OppA has chaperone-like activity on proteins which were chemically denatured. Our findings are in accordance with the chaperone-like behavior of another periplasmic substrate-binding protein, the so-called **m**altose-**b**inding **p**rotein (MBP or MalE). The maltose binding protein is capable of refolding denatured proteins and can bind maltose (Fox et al. 2001). Moreover, maltose is the natural substrate of the α-glucosidase enzyme, which is our target for refolding detection. Intriguingly, it does not only belong to the functional class of ABC transporters, but it also possesses chaperone-like activity (Boos and Shuman 1998). All of which makes it a good basis for comparison to OppA.

As observed in other cases, like DppA, the flanking domains (lobes) close over the cleft to deeply bury a large range of ligands (Tame et al. 1995; Quiocho et al. 1997). When compared to MBP, OppA also possesses a hydrophobic cleft for ligand binding, which is flanked by two lobes connected through a flexible hinge region. That common topological feature is thought to allow the OppA protein to function in a chaperone-like fashion as well. In contrast to true chaperones (e.g., GroEL and DnaK) however, MalE and OppA have a smaller cleft (Chen and Sigler 1999; Kobayashi et al. 1999; Tanaka and Fersht 1999). This fact leads to the question whether this reduced open space would hamper the chaperone-like refolding capacity of OppA?.

The maltodextrin-binding site constitutes an outspokenly hydrophobic cleft which makes it a likely target for interaction between fused polypeptides and MBP. This is not a far-fetched assumption because it is in good keeping with the following three published findings: First, authentic molecular chaperones (e.g., GroEL and DnaK) use hydrophobic clefts to heal denatured proteins by folding them back into their native states (Kapust and Waugh 1999). Secondly, the binding cleft possesses unusual conformational flexibility (Quiocho et al. 1997), adjusting its shape to the special (steric) requirements of the ligands in order to accommodate a larger variety of ligand peptides. Thirdly, proteins fused to MBP do not bind efficiently to amylose resin (Pryor and Leiting 1997), all of which could hint at their possible interaction with this hydrophobic cleft.

As a direct consequence, we changed the amino acidic residues Arg41 and Asp 42 for Alanine (R41A, D42A). The change in both amino acids affects the surface properties in the mutant OppA-R41A/D42A. In direct consequence the chaperone activity decreases compared to wild type OppA. This finding hints at the pivotal role of this surface patch for recognizing denatured substrate proteins.

The R41A and D42A mutations on the OppA protein lie in a surface patch near the cleft center (Fig. 3) and they exert a negative effect on the refolding of denatured proteins. The amino acid substitutions D419G and Y420G do not show effect on the chaperone activity of OppA (Fig. 4). These data suggest that the identified hydrophobic cleft is not related to the chaperone activity of OppA. According to literature (Richarme and Caldas 1997) the chaperone-like activity of OppA is not affected regardless whether OppA binds tripeptide substrate or not (i.e. closed or open states). Tanabe et al. (2007) reported that Asp419 belongs to the substrate binding residues and we chose to challenge this view. It turns out that it had no effects (i.e. negative control according to literature knowledge).

Moreover, it was expected that experimental conditions for denaturalization would differ as well. While α-GLD could be treated under basic conditions by urea at room temperature to yield reversible refolding, LDH was unable to show any activity under the same test conditions. Instead guanidine base treatment was used because enzymatic activity was gained to some minor degree in the presence of OppA. One of three mutants showed strong losses of refolding capcity in α-GLD and LDH alike (Fig. 4). As a most valuable asset, the treatments can be set in relationships to each other for result interpretation in a way that refolding activity can be indirectly inferred from the observed enzymatic activity differences. Passing the statistical significance tests for the in vitro activity measurements with success implies that the underlying refolding processes for the enzyme under scrutiny took place in a complete manner, i.e. compelling evidence is generated to proof that the proteins under scrutiny effectively work as refoldases. In cases with a p-value close to the threshold of 0.05 or even above, itcan be assumed that refolding took place but not in a perfect way. Finally, treatment comparison with wild type reveiled that some of our proposed mutation type OppA significantly affect chaperone activity.

The studied mutations constitute preliminary hints for the ongoing research in the field of chaperone-like activity of non-chaperone proteins. With a combined approach of computer models and laboratory experiments, we tried to find evidence about OppA’s possible participation in cell protein refolding. However, it would be too far-fetched to draw general conclusions at that stage about the underlying molecular mechanism of the refolding activity of OppA on another denatured enzymes. In the future more research has to be carried out to fully understand the underpinnings of OppA’s protein refolding capacities.

## Data Availability

Please contact to the authors for all request.
